# Integration of Micro/Nanobubbles into Biopolymer-Based Coatings: An Innovative Concept and Hypothetical Approach for Postharvest Preservation

**DOI:** 10.3390/foods15193509

**Published:** 2026-10-01

**Authors:** Nishant Kumar, Pratibha, Mahesh Mohan Shinde, Aneta Stajikj, Anka Trajkovska Petkoska

**Affiliations:** 1 Amity Food and Agriculture Foundation, Amity University, Noida 201313, India; 2 Centre of Management and Humanities (CMH), Punjab Engineering College (Deemed to Be University), Sector 12, Chandigarh 160012, India; pratibha91coo@gmail.com; 3 Food Technology, MIT-ADT University, Pune 412201, India; shindemahesh272@gmail.com; 4 Faculty of Technology and Technical Sciences, St. Kliment Ohridski University-Bitola, DimitarVlahov, 1400 Veles, North Macedonia; aneta.stajik@uklo.edu.mk; 5 Department of Materials Science and Engineering, Korea University, 145 Anam-no, Seongbuk-gu, Seoul 02841, Republic of Korea

**Keywords:** micro- and nanobubbles, biopolymer edible coatings, active or smart packaging, postharvest preservation, encapsulation, shelf-life extension

## Abstract

Postharvest losses are an enormous issue for food security and account for 30–40% of fruit and vegetable spoilage. Despite the potential of biopolymer-based coatings for fruits and vegetables as sustainable materials in comparison with traditional packaging, practical use is still limited due to poor barrier and mechanical properties and uncontrolled release of active components. Therefore, the proposed micro/nanobubble-integrated biopolymer edible coatings (MNB-BEC) framework builds on emerging studies of nanobubble-assisted biopolymer films and extends these findings toward a broader conceptual framework for postharvest edible coatings for minimizing food losses. The study focused on micro/nanobubble-integrated biopolymer edible coatings as an innovative strategy for advanced postharvest preservation. The proposed concept emphasizes the physicochemical properties, higher surface area, and gas–liquid mass transfer of micro/nanobubbles in biopolymer-based coatings to design multilayer coatings with higher functionality. This integration of biopolymers with micro/nanobubbles may help in enhancing barrier, mechanical, structural, and thermal properties of the film. In addition, manipulation of gases, including ozone (O_3_), CO_2_, O_2,_ and ethylene levels, resulted in the formation of an antimicrobial environment to reduce microbial spoilage and maintain the physicochemical properties and consumer acceptability of produce by retarding oxidative stress, moisture loss, and respiration rate. Innovative concepts and hypotheses focus on MNB-BEC, bubble formation techniques, types of insert gases, biopolymers, their characterization techniques, and applications. The major challenges, including interfacial stability, release kinetics, scalability, and regulatory and industrialization issues associated with the technology, were also discussed. With developments in colloidal formation, gas–liquid interfaces, and edible coating technology, the present concept offers a comprehensive conceptual framework for MNB-BEC as a sustainable and multifunctional packaging system.

## 1. Introduction

Fruits and vegetables are among the most perishable foods, making them especially susceptible to both quantity and quality loss during harvesting, handling, storing, transporting, wholesaling, and retailing. According to the Food and Agriculture Organization of the United Nations [1], the global Food Loss Index indicates that food losses between harvest and retail have been significant, with fruits and vegetables accounting for the commodity type that has recorded the highest losses, rising from 23.2% in 2015 to 25.4% in 2023. These losses result from high respiration and transpiration rates, mechanical injuries, microbial degradation, improper temperature and humidity control, and poor postharvest handling and infrastructure inherent in fresh produce. The FAO database on food losses and waste is a crucial data set for evaluating losses by commodity types, geographical regions, and supply chain stages [2]. Nonetheless, estimates of postharvest losses differ significantly depending on commodity types, geographical region, measurement techniques, and supply chain stages. Thus, context-specific and commodity-specific interventions need to be undertaken, not the generalization of postharvest losses. Food security is a critical threat and challenge due to continued food wastage and postharvest losses in fruits and vegetables at the global level. Microbial spoilage and mechanical and physical damage are major factors in postharvest losses, accounting for 40% throughout the supply chain [3]. To overcome postharvest losses, potentially effective postharvest handling and advanced sustainable preservation methods are required for shelf-life extension of fruits and vegetables [4]. Several postharvest management strategies, such as modified atmosphere packaging, controlled atmosphere packaging, edible/composite, and intelligent packaging, have been utilized for the preservation of postharvest quality of fruits and vegetables. Despite these technologies, edible coatings developed using naturally derived biopolymers, including polysaccharides, protein, lipid/wax, and their composites, are an efficient solution to control respiration and moisture losses in produce, which results from their barrier resistance, and minimize oxidative stress during storage and transportation. Edible coatings are biodegradable and eco-friendly in nature and can be used as alternatives to conventional and petroleum-derived plastic packaging. Nevertheless, conventional coatings prepared from natural biopolymers are often associated with the problems of poor coverage of fruit surfaces and poor ability to deliver bioactive compounds [5].

Polysaccharide-based coatings and edible films have been extensively studied to reduce moisture losses, modify gas transfer, and provide antimicrobial or antioxidant compounds to improve the storage life of fruits and vegetables [6,7,8]. Nevertheless, the performance of such systems is greatly influenced by the type of polymers used, formulation, coating thickness, and relative humidity. Hydrophilic polysaccharides and proteins tend to have good barrier properties against gases but have poor moisture-barrier properties, while lipids as coatings can enhance moisture-barrier properties but may affect the mechanical properties or the quality of coatings [9,10]. The structure–function relationship emphasizes the importance of methods that can effectively control coating microstructure and mass transfer. The application of micro- and nanobubbles (MNB) has become more prevalent as a gas/liquid system due to the small sizes of bubbles that have the ability to offer higher gas/liquid interface areas and affect gas dissolution, mass transfer, and physicochemical properties of aqueous systems [11,12,13,14]. Some recent review articles have highlighted the applications of MNB in food processing operations such as cleaning and disinfection, fresh produce washing, gas transfer, fermentation-related operations, extraction/separation, and material property modification [11,13,14].

The innovative concept of using micro- and nanobubbles integrated with biopolymers aims to improve the functional properties of edible coatings and films and to be potentially effective in the sustainable or controlled release of bioactive compounds [15]. This unique physicochemical behavior due to the presence of gas-filled structures allows for improved manipulation of fruit respiration and microbial safety in comparison with traditional coating formulas [16]. This integrated approach takes advantage of the potentially highly effective gas-transfer properties of the micro- and nanobubbles to optimize the mechanical and functional structure of the biopolymer films [6]. Regarding the incorporation of gas in the integrated micro/nanobubble biopolymer-based coatings (MNB-BEC) approach, the suggested MNB-BEC system takes into account the effects that micro/nanobubble characteristics, such as size distribution, interfacial properties, and gas composition/quantity, might have on the physical, structural, and mass-transfer properties of biopolymer-based coatings. It is assumed that these effects will largely depend on the chemistry of the polymer, microbubble retention during drying, interfacial stability, and storage. Moreover, the effect of certain gases used in the system on modification of the postharvest microenvironment is suggested, along with possible mechanisms of antimicrobial action and release of active compounds. Since there is insufficient experimental validation of the integrated MNB-BEC system, these proposed relationships are formulated as hypotheses and research directions [17].

Moreover, this multifunctional approach may help in addressing the bioactive compound delivery issue in the coating system and regulation of moisture and respiration in fruits and vegetables [18]. Additionally, although there are existing studies focusing on the potential effectiveness of composite packaging for gas-barrier performance, the impact of the use of matrices with bubbles on the mechanical strength of these layers is another area that needs to be explored for postharvest management [9]. The integration of the gas–liquid interface and the development of MNB-BEC systems offer a potential route for a sustainable approach as an alternative to chemical fungicides and pesticides to minimize postharvest losses by extending shelf life without affecting physiological and biochemical attributes [8]. Postharvest losses pose a significant threat to global food security and the environment due to microbial spoilage of perishable produce and need to be overcome [7,10,19]. Moreover, the existing packaging technology lacks the ability to address issues related to the balance of gas and moisture permeability in different conditions throughout the supply chain [20,21].

The rising consumer awareness and demand for organic foods lead to the need for the development of edible bio-based packaging materials as an alternative to synthetic materials [22]. Despite being a green substitute for synthetic and non-degradable plastics, films based on biopolymers often experience limitations in terms of their ability to potentially function effectively due to natural insufficiencies in the barrier properties against gases and the ability to withstand mechanical stress [23]. Biopolymeric matrices are unable to retain their structure in case of exposure to environments with high humidity levels, which makes it impossible to utilize them for potentially sustained release of bioactive components [24]. Moreover, there are numerous challenges associated with the stability and scalability of such films since current manufacturing technologies cannot provide homogeneous distribution of additives or structural cohesion of the films [25,26]. However, the incorporation of micro- and nanobubbles into the matrix allows for a new approach to control the structure’s porosity and increase the bioavailability of its components [27].

The scientific problem that is being considered in this review paper is connected to the interaction between three areas of research that have made much progress on their own. First, there is MNB gas–liquid interface engineering; secondly, there is an area of biopolymer-based edible coatings; thirdly, there is active and intelligent food packaging. It should be acknowledged that existing literature contains plenty of information about the process of MNB generation and stabilization in liquid phases as well as about the creation and usage of edible coatings. At the same time, there is no clear idea of what happens to MNBs when they become a part of biopolymer coatings and undergo drying and other transformations. The MNB-BEC approach thus does not assume that the introduction of bubbles will inherently enhance coating performance. Rather, the MNB-BEC approach considers the potential for MNBs to add an extra dispersed gas phase and gas–polymer interface whose influence depends upon the processing and formulation conditions. It aims to develop predictive relationships among the properties of the bubbles, polymer interactions, coating microstructure, transport and release properties, and their impact on postharvest functions. This point is particularly relevant since many benefits attributed to the use of MNB-BECs have not yet been experimentally proven.

This brings up research questions such as the following: Can micro/nanobubbles maintain their characteristic size distribution and spatial distribution through the processes of mixing, coating deposition, drying, and storage in biopolymer matrices? What is the role of polymer chemistry, viscosity, surface tension, charge at the interface, and the process of film formation in bubble retention and coating microstructure? Is there an impact of the inclusion of MNBs on oxygen, carbon dioxide, water vapor, or other gas transport in the final coating, and is the effect orientation-dependent on bubble loading and connectivity?

Testable hypotheses include the following: MNB incorporation changes the microstructure and interfacial characteristics of biopolymer coatings in a bubble-size- and concentration-dependent manner. Bubble retention during drying is significantly influenced by polymer viscosity, gas composition, coating method, drying rate, and environmental conditions. MNB incorporation alters the effective gas and moisture transport properties of the final coating; however, the direction and magnitude of this effect depend on bubble distribution, connectivity, polymer structure, and coating thickness. The incorporation of MNBs can modify active-compound release kinetics through changes in interfacial area, matrix structure, polymer swelling, and diffusion pathways. Gas-specific MNB-BEC formulations produce different effects on microbial growth and produce physiology because oxygen, carbon dioxide, ozone, hydrogen, and ethylene have different physicochemical and biological functions.

## 2. Novel Concept Overview

**Figure 1:**
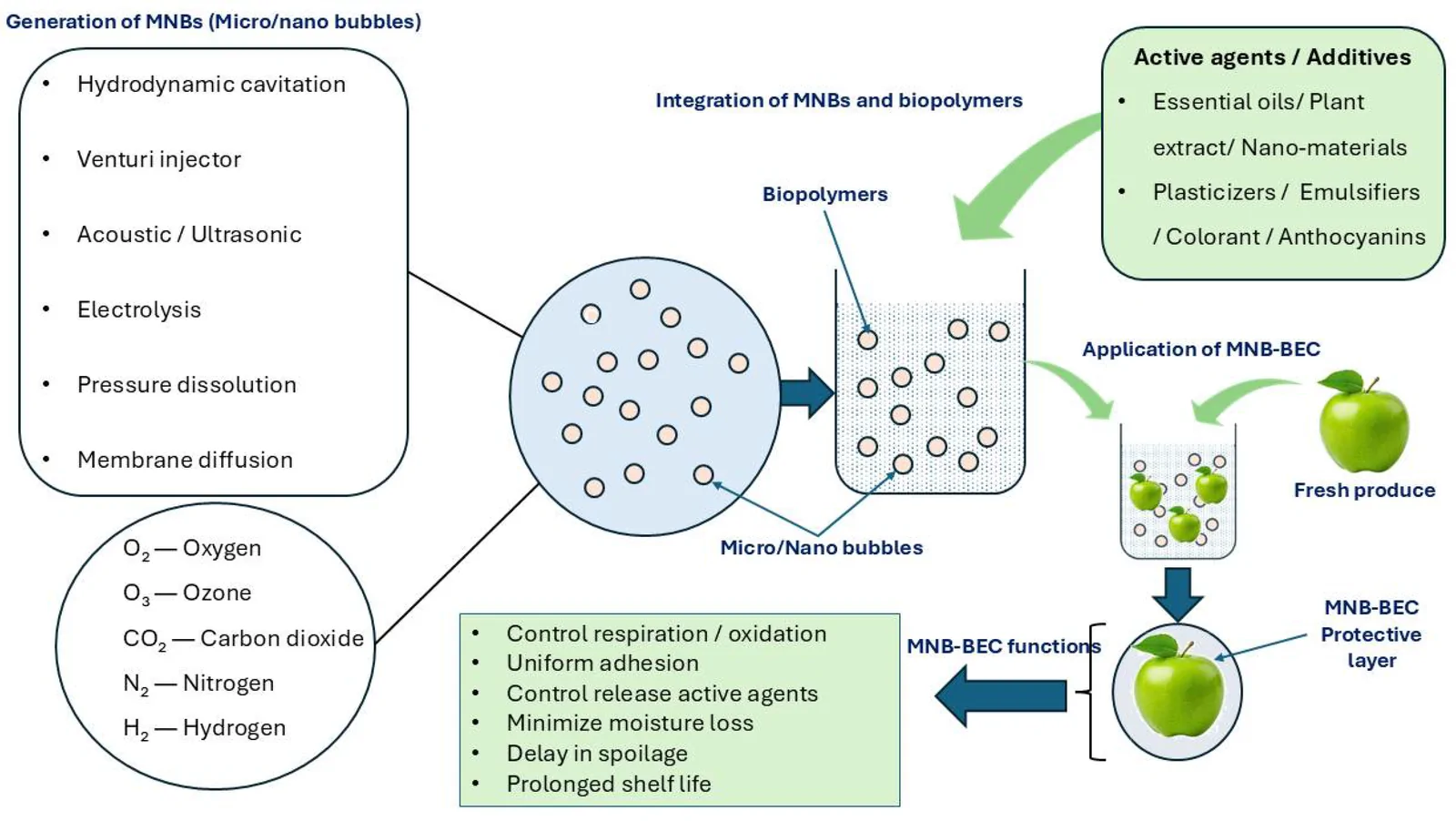
Proposed conceptual framework of micro/nanobubble-integrated biopolymer edible coatings (MNB-BEC) for postharvest management.

The proposed concept, the MNB-BEC platform, can offer an innovative solution to postharvest preservation using bubble-loaded materials as delivery systems for the release of active agents. The inclusion of gases improves and changes their efficient mass-transfer properties and interfacial characteristics. MNB-BEC technology could enable the creation of a dynamic barrier to improve the internal atmosphere, resulting in the inhibition of microorganism growth. MNB-BEC technology may facilitate an eco-friendly approach to extending the shelf life of commodities and maintaining their postharvest quality for a longer period. The proposed novel concept explores the basics of gas-loaded microcavities functioning within edible biopolymer matrices and their crucial contribution to optimal solute loading and thin film formation. In addition, the technology may be able to overcome poor adhesion and coating thickness inconsistency issues through the optimization of bubble concentration and size distribution. The use of the unique modification of bubble–biopolymer interactions improves the mechanical strength and moisture-barrier efficiency necessary for novel active packaging. The ultra-high specific surface area and unique charge distribution of these gaseous nanospheres may contribute to the improved interfacial interactions, which are superior to other modification approaches. Therefore, this research outlines the synergistic impact of gas–liquid interface stabilization within the polymer matrix, which acts as the blueprint for future studies on adaptive food packaging. Enabling the accurate stabilization of gaseous active components in structural voids, the approach tackles the volatility of gases, a limiting factor in the potential effectiveness of conventional packaging technologies. Therefore, the MNB-BEC approach offers a potential route to a platform that goes beyond passive film-forming, offering a new gas-responsive system adapted to the metabolic demands of horticultural fresh produce. Gas-containing micro/nanobubbles are incorporated within an edible biopolymer matrix. Figure 1 shows a potential graphical illustration of the proposed micro/nanobubble-integrated biopolymer edible coatings (MNB-BEC) framework as an innovative conceptual approach for postharvest preservation.

Innovation in encapsulation technology is crucial in addressing key deficiencies related to barrier potential effectiveness and functional delivery of the system, transforming conventional edible coatings into smart bioactive-responsive systems. The use of elevated pressure and longevity associated with these gaseous bubbles may result in the development of a potentially high-performing barrier, which may increase, under suitable conditions, the efficiency of gas–liquid mass transfer. This efficient transfer of mass may greatly increase, under suitable conditions, the scope of application of biopolymer films beyond mere physical barriers and make possible the potential stimuli-responsive delivery of nutraceuticals. This innovation minimizes the gap between the traditional passive barriers and intelligent active systems, resulting in the reduction of food waste after harvesting due to improved atmosphere management. Based on the theoretical foundation, this paper discusses the scientific breakthroughs in fluid dynamics and polymer science to design these smart gas-enriched systems. Given the synthesis of the available literature regarding the stability of micro- and nanobubbles in polysaccharide and protein matrices, the proposed comprehensive conceptual framework is necessary for developing intelligent preservation interfaces. Indeed, such trajectories of research are fundamental to further advancement of the field of food engineering since they allow for tackling the problem associated with the development of new colloidal structures from underexploited edible biopolymers. In this sense, the integration of the concept of MNB-BEC is a new research hotspot connecting the field of passive barrier solutions and highly responsive packaging solutions.

### Feasibility of the MNB-BEC Concept

The feasibility of incorporating MNBs into the biopolymer edible coating would depend on the ability of the bubbles to maintain their stability during the processes of mixing, coating application, and drying. However, the stability of the bubbles in the precursor dispersion is no guarantee that these bubbles will survive in the resulting coating, since alterations in viscosity, surface tension, evaporation rate, polymer concentration, and the drying process might favor bubble dissolution, migration, coalescence, and/or breakage. Thus, the suggested MNB-BEC concept should be viewed as process-dependent and require optimization of bubble size and concentration, gas mixture, polymer type, coating technique, and drying conditions. The evidence based previous studies on the development of system with integration of micro/nanobubbles and their functional properties are depicted in Table 1.

## 3. Micro/Nanobubble Technology: Properties, Functions, Bubble Generation Methods and Gases and Mechanisms

The key features of microbubbles and nanobubbles are the high surface-area-to-volume ratio and the stability of gas dissolution, owing to the inhibition ll size of the bubbles, normally in the order of tens of nanometers to several micrometers [33]. These properties allow for an extended presence in liquid media and favor the formation of reactive species, which may help in microbial inhibition [34]. Zeta potential offers a potential indicator of colloidal stability and electrostatic interactions in the precursor dispersion; however, it should not be interpreted as direct evidence of adhesion or mechanical reinforcement in the dried MNB-BEC. Final-coating adhesion and mechanical performance depend additionally on polymer–bubble interfacial chemistry, polymer chain organization, drying history, bubble retention and coating–substrate interactions [35]. The inherent buoyancy and peculiar surface charge of gas-containing structures mean they can be employed as reinforcements in polysaccharides and protein-based matrices, thus avoiding the permeability constraints of conventional biopolymer films [36]. The incorporation of gases in coatings allows them to turn their morphological structure into a complex colloidal system capable of controlling the mass transfer rate and preventing spoilage of produce [37]. By taking advantage of these modifiable colloidal properties, MNB-BEC systems may provide a structured environment for incorporating volatile bioactive agents such as essential oils into biopolymer matrices to increase their antimicrobial activity without sacrificing sensory qualities, although the location of the active compounds and the mechanisms governing their retention and release require experimental validation [38]. In addition, the decreased interfacial tension resulting from the use of these gaseous compartments may contribute to more homogeneous dispersion of active substances and may increase protective properties beyond the capability of traditional film systems [39]. The structural modification transforms biopolymer matrices from passive structures to active structures that protect against pathogen growth and maintain higher nutritional and physicochemical properties of produce [40]. Moreover, this innovation may help address the problems associated with traditional gas–liquid transport due to the poor solubility and fast decomposition of active substances [41]. Incorporation of such gaseous cavities into the biopolymer matrix takes advantage of the special hollow shell–core structure of the nanobubbles to increase the stability and functional availability of the material [42]. Moreover, the high internal pressure and gas–liquid mass transfer mechanisms in bubbles help to achieve the controlled release of certain gas cores in an amount sufficient for the specific metabolic needs according to the product [38]. The gaseous inclusions work as internal structural modulators, reducing the viscosity and potentially preventing the process of aggregation of structural elements in protein solutions, thereby allowing for an even distribution of coatings [43]. Moreover, incorporation of such bubbles in biopolymer networks allows for creating tortuous paths for moisture and gas diffusion to improve the barrier properties of film packaging [44]. The combination of unique gas features with biopolymers helps in the development of a hybrid system with optimized structural stability and release kinetics [38,45].

This integration of materials science and bubble technology offers a new approach for mitigating mechanical instability, mass transfer, and surface tension to enhance the wetting properties of the film on the skin surface of fruits [28,46]. It also creates an eco-friendly path to modifying the physicochemical environment at the food interface. It correlates with existing approaches to solving the problems associated with conventional films, including excessive moisture transmission and mechanical fragility, using colloidal reinforcement to obtain better results [47,48]. In addition, the hollow core-shell construction of such inclusions makes extremely effective mass transfer possible, and gas–liquid interactions [49] can be utilized to control the breathing rate of fresh produce. Creating a potential control system for the atmospheric microenvironment helps in reducing oxidative stress and delaying the process of senescence in agricultural produce [3,5]. Therefore, this technology also addresses the gap between the barrier of films and the encapsulation of active components with sustainable release [50,51]. Therefore, the advanced packaging materials and systems minimize the issues associated with preserving fresh produce through the optimization of the moisture and gas permeability properties of edible films [52]. The novel combinations constitute a significant breakthrough from traditional coatings due to their use of colloidal science to overcome the perpetual problems associated with mechanical strength versus gas-barrier potential effectiveness [53]. This approach can create “intelligent” packaging to reduce food and postharvest losses by reacting to the postharvest environment of produce and reducing the use of chemical and synthetic preservatives [54]. By way of transformation from passive systems to these more active, gas-modulated structures, the MNB-BEC concept represents an attempt to bridge the gap between traditional hydrocolloid systems and more advanced materials based on nanocomposites [16].

### 3.1. Bubble Properties and Characteristics

Both micro- and nanobubbles are characterized by their hydrodynamic size ranges. Microbubbles range between 10 and 50 µm, whereas nanobubbles are under 1 µm, resulting in a high surface-area-to-volume ratio. MNB stability should not be attributed solely to high surface charge or zeta potential. Bubble stability is a multifactorial property influenced by bubble size distribution, gas composition, dissolved-gas concentration, pH, ionic strength, temperature, dissolved organic matter, surfactants, polymer composition, viscosity, interfacial rheology, and the bubble-generation method. Zeta potential can provide useful information about the electrostatic characteristics of the bubble interface, but it should not be considered a standalone predictor of bubble persistence in biopolymer formulations [12,55,56]. In MNB-BEC systems, the stability of bubbles within the polymeric matrix is therefore expected to depend on both bubble-interface characteristics and bubble–polymer interactions and requires experimental verification.

Accordingly, a high magnitude of zeta potential in the dispersion state should not automatically be interpreted as evidence of long-term stability after incorporation into a biopolymer coating. The relevant parameter for MNB-BEC development is the retention and structural integrity of the dispersed gas phase throughout mixing, coating deposition, drying, and subsequent storage [55,56]. The “self-pressurization” behavior of nanobubbles has been discussed in relation to Laplace pressure, gas dissolution, and interfacial phenomena; however, the mechanisms responsible for the apparent long-term stability of bulk nanobubbles remain under active investigation [55]. Any proposed formation of reactive oxygen species should also be considered gas- and process-dependent rather than an intrinsic consequence of all MNBs. The presence of these gas bubbles helps in improving the barrier properties of the film due to the creation of a tortuous path that may prevent the loss of excess moisture and oxygen [57]. Due to the large specific surface area of these gas bubbles, composite films with tunable porosity can be made to act as advanced “gas switches” that may modulate the O_2_ and CO_2_ exchange depending on the needs of horticultural products [19]. Functional distinctions among candidate gases are shown in Table 2.

Gases in the MNB systems should not be seen as equivalent agents to each other. This might be due to the effects that are influenced by the gas solubility, the oxidation–reduction reaction, the concentration, the period of contact, and interaction with the food matrix. In this case, ozone is distinct from oxygen, carbon dioxide, and hydrogen due to the oxidative nature of ozone.

### 3.2. Bubble Generation Methods

**Figure 2:**
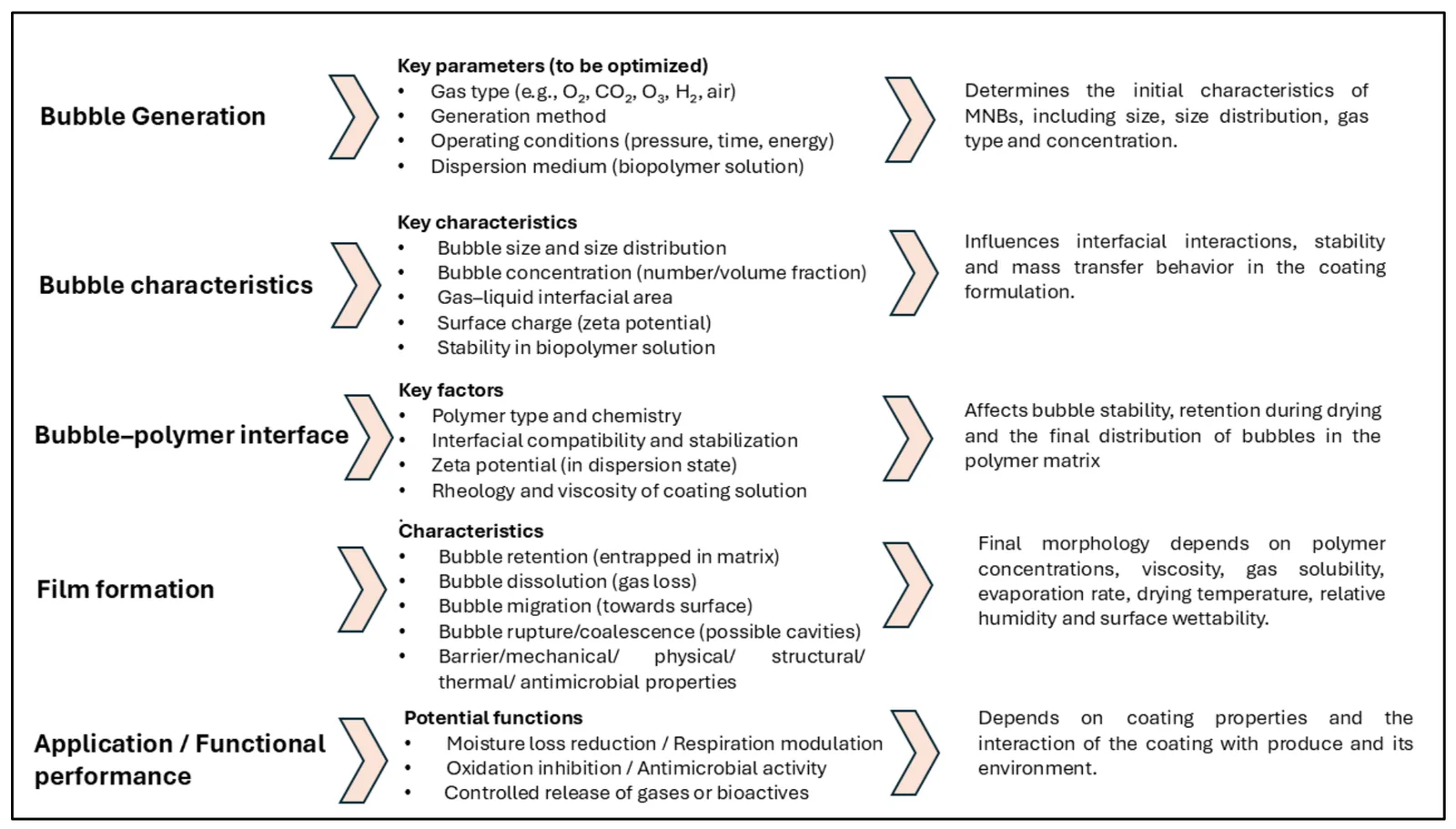
Proposed Structure–Property–Function Relationships in MNB-BEC Systems.

Based on analogous systems, it can be hypothesized that the process of generating these bubbles is carried out using the methods of hydrodynamic cavitation and pressurized saturation, which rely on the use of localized shear forces and pressure drops to create stable gaseous nucleation sites within liquid precursors [58]. The use of ultrasound radiation and microfiltration based on membranes may offer a potential route for better control over the bubble size and thus allow the design of bubbles that fit morphological needs in case of different matrices made of biopolymers [59]. Based on analogous systems, it can be hypothesized that rotational devices could also be used for controlled shear and gas introduction to generate stable bubbles, and the choice of gas phase, such as oxygen, carbon dioxide, or ozone, is critical for the functionality of such systems [60]. The use of oxygen-filled nanobubbles has been proven to modify interfacial redox conditions, while hydrogen-containing bubbles could function as scavengers that may prevent oxidation processes [61]. On the other hand, ozonized bubbles take advantage of their strong oxidizing nature, disrupting the membranes and enzyme functions of microorganisms to present a notable advance in comparison to passive antimicrobial materials [24,45]. This control of interface gas composition may permit the use of intelligent biopolymer networks to form dynamic barriers, overcoming the drawbacks associated with moisture-sensitivity and fragility of conventional protein and polysaccharide coatings [62,63]. Through careful manipulation of the gas-filled architectures, intelligent MNB-BEC systems can react intelligently to stimuli under storage conditions, thus addressing the balance between breathability and protection in conventional edible films [64,65]. The choice of fabrication methodologies such as membrane infusion or pressure-controlled electrolysis allows for potential manipulation of bubble populations for stability in different food processing conditions, including heating and mechanical stress [66]. Proposed Structure–Property–Function Relationships in MNB-BEC Systems are shown in Figure 2.

### 3.3. Proposed Active Compound Release Mechanisms in MNB-BEC Systems

The release of the active compounds from MNB-BEC systems can happen using multiple complementary mechanisms. The first one is diffusion of the active compound through the polymer matrix to its surface and then to the surrounding food matrix. The second mechanism can be dissolution of bubbles when gas dissolved in bubbles transfers into the aqueous phase, thus decreasing the size of bubbles and changing matrix structure. The third mechanism is a bubble rupture or collapse, which changes porosity and interface characteristics and may help active compounds to be exposed or released. The fourth mechanism is the swelling of the polymer due to water absorption, increasing the mobility of the polymer chains and thus increasing the diffusion pathways. The fifth mechanism is matrix degradation over time that will help to release active compounds entrapped in the polymer matrix. The sixth one is the influence of external stimuli on the polymer-compound interactions, stability of bubbles, swelling, or matrix structure, which provides stimulus-responsive release.

### 3.4. Structure–Property Relationships in MNB-BEC Systems

Table 3 shows the structural and properties relationship between the micro/nanobubble and biopolymer-based coating system.

The interfacial characteristics of micro/nanobubbles (MNBs) can be related to their characteristic diameter using the surface-area-to-volume relationship for spherical bubbles:

A/V = 6/d

where A/V is the surface-area-to-volume ratio and d is the bubble diameter. Thus, as bubble diameter decreases, the interfacial area available per unit volume of gas increases, which provides a theoretical basis for the high gas–liquid interfacial area associated with smaller bubbles [67]. This relationship is particularly relevant when considering gas dissolution and interfacial mass-transfer processes in MNB-containing formulations.

Gas transport through a polymeric coating can be described using Fick’s first law:

J = −D * (dC/dx)

where J is the diffusive flux, D is the diffusion coefficient, and dC/dxdC/dx represents the concentration gradient across the coating. Under the solution–diffusion framework, gas permeability can be expressed as follows:

P = D × S

where P is permeability, D is the diffusion coefficient, and S is the solubility coefficient of the gas in the polymer matrix [68,69].

For the proposed MNB-BEC system, incorporation of bubbles may alter the effective diffusion pathway, local polymer free volume, gas–polymer interactions, gas solubility, and interfacial resistance. Accordingly, bubble incorporation can modify the effective diffusion pathway, local solubility, and interfacial resistance, but the direction and magnitude of the resulting change in permeability cannot be assumed a priori. The final transport behavior is expected to depend on bubble size and distribution, bubble volume fraction, connectivity, bubble retention or loss during drying, polymer structure, coating thickness, and gas–polymer interactions. Therefore, the influence of MNB incorporation on the permeability of the final biopolymer coating requires experimental determination rather than being inferred solely from the high interfacial area of MNBs.

### 3.5. Barrier Function Versus Gas-Transfer Function

Integration of micro/nanobubbles (MNBs) into the biopolymer coating might affect the process of gas transportation through either alteration of the diffusion route inside the polymer matrix or provision of a gas–liquid interface for gas dissolution/exchange. These effects should be analyzed independently, without presumption that incorporation of MNBs will increase both the barrier properties and the efficiency of gas transfer simultaneously.

#### 3.5.1. Barrier Properties

If bubbles are dispersed and integrated properly in the dried biopolymer coating, they might be seen as dispersed domains within the polymer matrix, which can disrupt the continuous diffusion route. Therefore, substances such as water vapor or oxygen would be forced to move along a more complicated path.

Dispersed bubble domains may increase the tortuosity of the diffusion path and decrease diffusivity, which may decrease permeability.

Nevertheless, the impact depends greatly on the final microstructure. If bubbles are not well dispersed, are over-concentrated, interconnected, or leave pores behind during the drying process, they might just lead to preferred mass-transfer channels and result in high permeability.

#### 3.5.2. Gas-Transfer Capability

In wet precursors or at the gas/liquid interface, MNBs have a relatively high interfacial surface area owing to their tiny size. The interface can enable gas/liquid exchange, which includes the dissolution of the gas phase within the surrounding liquid.

Tiny bubbles with a high gas/liquid interfacial surface area per unit gas volume may have a high potential for gas dissolution/exchange.

Note: That gas/liquid transfer in the precursor does not always imply high gas permeability in the dry film. The two processes happen under different physical forms and operate on different principles.

#### 3.5.3. Connection Between the Two Functions

MNB-BECs can be perceived not as a system where both characteristics automatically grow at the same time but as a system balancing gas-transfer and barrier functions.

Consequences of the introduction of micro/nanobubbles to gas transport in the resulting coatings are to be assessed from the perspective of two different and potentially conflicting functionalities: the mass transfer of gases between the gas and the liquid phases in the coating composition and the barrier properties of the formed dried coating. On the one hand, in the initial dispersion, the high specific interfacial area of MNBs might promote gas solubilization and exchange between the gas and liquid phases. On the other hand, after the formation and drying of the film, remaining and well-dispersed bubbles could change the structure of the polymer and increase the tortuosity of diffusion paths, thus lowering the permeability of the coating. At the same time, an excessive bubble concentration, bubble merging, connections, or losses during the drying process can result in pore formation or preferential paths and thus increase mass-transfer processes. Hence, better gas–liquid mass transfer and increased barrier properties cannot be viewed as always complementary phenomena. The final transport properties are expected to be determined by the bubble size and volume fraction, their distribution and connectivity, gas solubility, polymer density, interfacial resistance, coating thickness, and retention during the drying process.

### 3.6. Antimicrobial Activity and ROS-Mediated Mechanisms

The antimicrobial action of MNBs should be regarded as gas- and process-specific instead of being attributed intrinsically to all types of MNBs. This process may depend on the type of gas, the method of generating bubbles, the working conditions, bubble size, bubble concentration, gas–liquid interface properties, dissolved gas concentration, and physical and chemical properties of the medium surrounding the bubbles [12,40]. The proposed mechanism may be expressed in the following way: gas type → bubble-generation conditions → bubble properties and gas–liquid interface → dissolved gas and/or cavitation-associated processes → generation of reactive oxygen species → damage to microbial cells → antimicrobial action. Ozone-loaded MNBs represent the most illustrative case since ozone is an oxidizing agent and ozonated MNB systems possess antimicrobial properties [40]. Reactive oxygen species such as hydroxyl radicals can form in ozone MNB systems during reactions at the gas–liquid interface and while bubbles shrink or implode. This means that the antimicrobial action of ozonated MNBs is determined by both the chemical properties of ozone and the gas–liquid interface properties of MNBs.

In comparison, oxygen/air-containing MNBs could affect oxygen availability and, under certain physiological and chemical conditions or activations, cause ROS formation, but ROS production cannot be explained by the presence of MNBs alone. New information suggests that the biological activity of nanobubbles depends greatly on the gas component and working conditions. Carbon dioxide-containing MNBs could affect microbial growth through carbon dioxide dissolution, pH changes, gas availability, and corresponding physicochemical effects without resorting to a common ROS pathway. The hydrogen-containing MNBs can be considered in terms of redox and antioxidant effects, not as antimicrobial agents [12].

Therefore, in the suggested MNB-BEC system, antimicrobial activity should be seen as a functional hypothesis that needs to be tested through specific gas-based, process-based, and matrix-based experiments, which will examine the effects of dissolved gas, ROS/oxidative capacity, and microbial reactions. Antimicrobial activity from MNBs, therefore, should be seen as gas- and process-specific and not an inherent characteristic of nanobubbles [12,40].

## 4. Biopolymer-Based Edible Coatings

Biopolymers such as polysaccharides, proteins, and lipid/wax are derived from plant, animal, and microbial sources; these matrices are non-toxic and biodegradable in nature [63]. Among such materials, chitosan is one of the most promising primary biopolymers owing to its ability to form films and its natural antibacterial properties. Thus, chitosan can be used to develop a protective film, which may also allow for controlling microorganisms during the postharvest preservation process. Alginate and pectin are also applicable, as they can form continuous films and are commonly applied in edible films. Starch offers a potential route for good film-forming properties, encapsulation, and emulsification capabilities; nevertheless, films made from starch need to be strengthened due to low mechanical properties and barrier capability. Similarly, polysaccharide-based films are potentially effective oxygen and CO_2_ barriers but can be less resistant to water vapor because of their hydrophilicity [27,70]. In the case of coating systems based on micro- and nanobubbles, chitosan can be considered as a main polymeric matrix for forming films, whereas alginate, starch, or pectin can be regarded as alternative matrices or blending polymers. The chosen polymer needs to be compatible with the technique of bubble generation and should ensure uniformity, adhesion, stability, and edibility of coatings. Moreover, the polymeric matrix needs to be able to encapsulate bubbles and distribute active ingredients in coatings without any influence on sensory characteristics of the final product. However, the potential effectiveness of such films depends on the regulation of gas and water transfer. In fact, the restrictive properties of such films are necessary to reduce oxidation and dehydration [71]. For example, coatings based on polysaccharides suggest permeability to O_2_ and CO_2,_ which are essential for slowing down the process of senescence and could influence the internal atmosphere [72].

MNB stability and entrapment in edible films made from biopolymers are likely to be affected by the physical properties of the coating matrix and film formation method. While polymer viscosity can limit bubble movement and collisions between bubbles, too much viscosity might impede proper bubble dispersion and coating formation; surface tension and charge at the interface are expected to affect bubble formation and coalescence. This depends on the matrix and can also be altered by other factors such as polymer concentration, pH, ionic strength, surfactants, and other ingredients [12,14]. Even the coating process itself can affect the bubbles’ ability to remain inside the coating matrix. Immersion is done through dipping, withdrawal, and drainage. Spraying is accomplished through atomization and shear, while casting entails solvent evaporation and increased matrix concentration through drying. All these processes have the potential to cause changes in the bubbles, such as redistribution, dissolution, coalescence, or collapse [9,73]. As such, MNB stability in the initial solution does not guarantee stability within the final dried coating film, and characterization of the bubble size distribution/concentration should be done both prior to and post coating formation. From an industrial perspective, other considerations for the use of edible coatings include cost of ingredients and production, consistency, coating thickness control, sensory acceptability, regulations, and compatibility with current equipment [9]. These suggest that MNB-BEC development would entail the simultaneous optimization of all the above aspects and not just the bubble stability itself.

## 5. Rationale for MNB-BEC Integration

The integration of micro/nanobubbles in biopolymer-based edible films helps overcome certain drawbacks of traditional films, such as lack of water stability and suboptimal delivery of active antimicrobial agents [74]. Due to their incorporation, the matrix allows for prolonged release of the entrapped substances and may improve its barrier properties by providing protection from moisture sensitivity, which is usually a problem for starch- and polysaccharide-based films [75]. The present literature shows that biopolymers are efficient gas exchange regulators but lack mechanical stability and the ability to create a moisture barrier that would protect highly perishable products [76].

The introduction of gas bubbles into the polymer matrix generates another phase, which is used as a structure stabilizer, leading to increased tortuosity of the diffusion path for water [77]. Moreover, the gas bubbles inside the structure create internal barriers that disrupt the crystalline structure of the polysaccharide matrix and thus make coatings more flexible [78]. The proposed MNB-BEC design does not suggest that active compounds are inherently situated in the gas phase of the bubbles. The position of active components could be in the polymer matrix depending on the design approach, or at the bubble–polymer interface, or even in the regions of the polymer phase adjacent to the gaseous structures inside. Thus, the function of MNBs could involve the modification of interfacial layer properties, the microstructure of the polymer matrix, the release pathway, or water-induced transfer, but MNBs may not necessarily act as reservoirs for the active compounds. Release of the compound could happen due to diffusion through the polymer matrix, relaxation of the polymer material, gas dissolution or bubble collapse, desorption from the interfacial layer, and gradual degradation of the matrix. Relative humidity, temperature, pH, and osmotic pressure could influence these processes as well. Such approaches to release mechanisms are suggested now for the MNB-BEC design and need validation through release kinetics studies, microscopy, migration, and diffusion modeling.

Incorporation of MNB into a delivery vehicle can thus provide a microstructure that enables the controlled distribution and transport of active agents via bubble–polymer interactions and modifications to the matrix. It is important to note that the protection and stimulus-triggered release of bioactive compounds are still only hypotheses for MNB-BECs, and those alterations to pH, osmolarity, humidity, and temperature may impact bubble stability, polymer hydration, interfacial interactions, and diffusional pathways; the extent of such impact must be investigated experimentally. Justifications for using MNBs in edible coatings based on biopolymers would be to incorporate an extra gas–liquid interface that would affect the structure, gas transfer, interfacial interactions, and functional compound distribution in the coating rather than incorporating just another active compound. Nonetheless, incorporation of MNBs can result in negative consequences such as bubble coalescing/collapsing during processing, formation of pores or preferential diffusion pathways, decrease in mechanical strength, uneven coating structure, loss of gases during drying, and extra cost associated with processing. Hence, the key hypothesis is that the incorporation of MNBs causes significant differences in the structural, barrier, mechanical, gas transfer, and release properties of biopolymer-based edible coatings relative to the same coatings without any MNBs, where the extent of differences is affected by the size and concentration of MNBs, gas composition, polymer matrix, method of coating, and storage conditions. H_0_: There are no significant differences between the properties or postharvest performance of the coatings containing MNBs and those without MNBs. Validation would involve a sequential process of (i) generation and characterization of the MNB, (ii) investigation of the compatibility and stability of the polymer-bubble system, (iii) coating generation and bubble retention assessment, (iv) characterization of the coating morphology, thickness, mechanical and barrier properties, (v) drug loading and delivery studies, and lastly (vi) validation of the MNB-BEC on selected fruits/vegetables under specified storage conditions followed by sensory, safety, scalability, and economic evaluation. Since the available evidence of MNB is more compelling in relation to liquid foods and processes compared to dried biopolymer coatings, the proposed roles of MNB-BEC, which include enhanced barrier behavior, controlled release, antimicrobial effect, and extended shelf life, should be considered hypotheses.

## 6. Characterization and Function of MNB-BEC Systems

The evaluation of these systems demands state-of-the-art analytical methods, such as nanoparticle tracking analysis and cryo-scanning electron microscopy, to determine the bubble size distribution and interfacial morphology in the biopolymer matrix [79]. Moreover, the study of structure–function relationships in MNB-BEC systems requires rheology and mechanical stress tests to make sure that the presence of a gaseous dispersed phase may improve the tensile strength and barrier efficiency without sacrificing the viscoelastic properties of the film. The standardization of the protocols is necessary to perform reproducible comparative evaluation and measure the uniformity of dispersion and gas-transfer efficiency, and thus assess the stability of these systems under different conditions [13,14]. The development of benchmarks will contribute to further implementation of laboratory-scale prototypes into industrial use and help to satisfy growing demands of the market for clean-label preservative technology. Further investigation should focus on the assessment of the potential effectiveness of these systems on different fruit varieties to prove that the inhibition of the oxidation process in fruits by antioxidant release is efficient during prolonged storage periods. By investigating physicochemical characterization and oxidative stability tests, this theoretical framework helps to fill the existing research gaps concerning the efficiency of bioactive delivery within complex food matrices [80]. This approach will help establish a standardized method of formulating “generally recognized as safe” bioactive carriers that possess the necessary mechanical strength and may enhance functionality to preserve food products [23,81]. In this context, Table 4 presents MNB-BEC systems and their potential characterization by use of standard methods.

Nevertheless, the MNB-BEC concept remains subject to substantial uncertainties regarding bubble retention during film formation, the relationship between bubble structure and gas/moisture permeability, gas-specific functionality, active-compound release, long-term stability, sensory acceptance, safety, and industrial scalability. These uncertainties emphasize the need for systematic experimental validation before any performance or shelf-life benefits can be considered established.

### 6.1. Structural Characterization

The key role in this context is played by microscopy, particularly cryo-scanning electron microscopy, which is crucial to determine the spatial distribution and morphology of the bubbles in the polymer matrix [85,86]. The spectroscopic studies, for instance, using Fourier-transform infrared spectroscopy, are used to validate the bubble–coating interactions and confirm their effectiveness based on changes in the vibration of functional groups, which is a sign of proper interfacial stabilization of the gas [87,88]. Also, rheological assessment of the new composite materials is needed to evaluate the viscoelastic properties and structural stability of the new materials to provide sufficient mechanical performance of the high-performance packaging films [89,90]. Stable benchmarks are crucial to overcoming the problems with reproducibility of laboratory-scale biopolymer films [91]. Additionally, rigorous stability testing under simulated storage conditions is recommended due to the recent advances in active packaging matrices [92,93]. Empirical validation is important for addressing the challenges that come with the limitations present in biopolymer-based barriers due to their inability to possess the required flexible structures [94,95]. This conceptual model can be used as a potentially effective guide for improving the effectiveness of active packaging by making use of MNB-BEC interactions [96,97].

### 6.2. Stability Profiling

The study of bubble permanence in these biopolymer films over time is very important, as the gradual fusion or diffusion of these entrapped gases could greatly affect the ability of the coating as a potentially effective protective barrier [98]. Therefore, it is vital to subject the system to different environmental stresses such as temperature and relative humidity variations to see the effects that these have on the bubble structures inside the coating [99]. Prospective research on the shelf life of these MNB-BEC systems in different storage environments is important to ensure that the structural quality of the film is not affected by oxidative damage from bubbles or loss of gas from bubbles [39]. The linkage between these changes in the structure and the gas permeability properties of the film is crucial in establishing the potential effectiveness of the active packaging [100]. Additionally, integrating such findings into standardized metadata reporting and harmonization of units, as called for in recent studies on sustainability of barrier systems, will enable better comparison with industry-related packaging [101]. Additionally, future research must include real-time applications to validate such findings by testing the performance of the packaging material in reducing spoilage throughout the supply chain [102]. Moreover, the migration study of active agents in the packaging system along with their stability under real-time application also needs to be investigated [103].

### 6.3. The Destinies of Bubbles in Film Formation and Drying

MNBs can experience different physical changes during film formation and drying that depend on the nature of the polymer matrix and the drying conditions. There are four possible fates for MNBs. Retention of bubbles: The bubbles remain trapped in the polymer matrix and become an integral part of the structure of the dried film. Dissolution of bubbles: Gas contained in the bubbles is dissolved in the aqueous phase and may be released during film drying, leading to elimination of bubbles. Bubble migration: During the evaporation of water and structural changes in the film, the bubbles can migrate to the surface of the coating or any other low-resistance region. Bursting/merging of bubbles: The bubbles can burst, merge, and coalesce during film drying, resulting in the formation of some pores in the film. Therefore, the final morphology of the dried film and bubble distribution depend on several parameters, including the concentration and viscosity of the polymer solution, gas solubility, size distribution of bubbles, evaporation rate, temperature of drying, relative humidity, wettability of the surface, and interfacial stability.

### 6.4. Functions of MNB-BEC

The main operating principle of MNB-BEC technologies may rely on the controlled management of gas permeability and bioactive compound release to influence the metabolic environment of fresh fruits and vegetables [104]. Micro/nanobubbles offer a high interfacial surface area, which helps to enhance solubility and mass transfer of natural antioxidant coatings, thus enabling better management of the release kinetics required for different commodities [102]. In this manner, both principles provide potentially effective means of preventing postharvest aging through optimized gas exchange and may prevent internal browning of perishable fruits and vegetables [39,105]. Moreover, the accurate regulation of the respiratory condition using such gaseous interfaces could prove to be potentially effective method for enhancing the shelf life of very perishable produce by reducing its ripening due to ethylene [36]. It can also act as an adaptive barrier through which the presence of the bubbles will ensure maximum barrier quality against oxidation and enzymatic degradation [106].

#### 6.4.1. Physiological Regulation

This integration of gaseous barriers helps regulate the atmosphere around the commodity, thereby helping to inhibit the rates of respiration and ethylene formation [107]. These barriers help create an altered atmosphere through the control of oxygen transfer and may prevent oxidative stress and enzymatic browning in climacteric fruits [108,109]. Additionally, these combined barriers may help prevent senescence-related metabolite formation, which helps maintain the structure of the fresh produce [110].

#### 6.4.2. Shelf-Life Extension

The integration of micro/nanobubbles within the biopolymer matrix could enable the regulation of the inner gaseous atmosphere in such a way as to prevent the ripening process and reduce senescence-induced loss of quality in fruits and vegetables [111]. MNB-BEC systems can improve the water- and gas-barrier properties of the film and be more functional than conventional packaging [5].

## 7. Conceptual Contribution

It was previously proven by earlier studies that nanobubble-mediated processing techniques can affect biopolymer-based film systems. For instance, the dispersion of thymol-loaded zein nanoparticles in gelatin films through the process of nanobubble water treatment was investigated; results showed influences on the dispersion of nanoparticles, intermolecular interactions, mechanical properties, and water-vapor permeability. Hence, the innovation in this study does not consist of introducing for the first time the use of nanobubble-related systems in a biopolymer film system. The innovation is the conceptual integration of the bubble characteristics, gas chemistry, interfacial phenomena, transport mechanisms, active release, and postharvest preservation in a unified MNB-BEC model. This study proposes a conceptual framework for integrating micro/nanobubble-mediated interfacial and gas-transfer phenomena into biopolymer-based edible coatings for postharvest applications. The proposed framework aims to incorporate micro/nanobubbles into biopolymer-based edible coatings, thus creating a new theoretical basis for dynamic, interface-mediated preservation. The proposed concept offers a potential route to a solution to the current issue of conventional packaging and chemical preservatives. It offers a potential route to solutions by offering an approach that goes beyond static and passive coatings, thus making environmental modulation possible. Thus, through the creation of a nexus between bubble-mediated gas modulation and sustainable barriers, this theoretical model becomes instrumental in making the transition away from existing packaging possible. Future empirical work will be concentrated on studying the interfacial stability of MNB-BEC at different levels of humidity and temperature, thus overcoming the gap that exists between theoretical and practical aspects of the topic.

## 8. Novelty and Future Perspective

The innovation of MNB-BECs comes about through their evolution from conventional barrier materials to active interface materials, which can modulate the postharvest microenvironment at the micro- and nanoscale. This innovative approach brings about a transition from the use of conventional packaging to active packaging technology through the manipulation of gas–liquid interfacial effects to control mass transfer, improve barrier function, and increase the shelf life of fresh produce. Regarding the development of bio-packaging materials, MNB-BEC technology helps overcome the trade-off between mechanical performance and gas-moisture barrier properties of multifunctional coatings. The integration of micro- and nanobubbles into the biopolymers brings about an innovative structural and functional strategy in which gas interfaces are used to affect the coating structure and transport properties.

Future research needs to be directed towards understanding the interaction between bubble interfaces and biopolymer networks and their effect on the loading, stability, and release rate of the actives. The combination of fluid dynamics, colloids, polymers, and food science can further help in optimizing the gas-loading process and development of scalable MNB-BEC systems. These developments can lead to the discovery of biodegradable options for plastic packaging derived from petroleum. The perspective of research on the MNB-BEC development process should be based on rigorous validation with real-time applications. Therefore, future research needs to move beyond the current stage of the characterization of MNB-BECs at laboratory scale to the study of this system with real fresh produce, with the proper evaluation of its potential effectiveness, stability, safety, migration issues, regulation, and scalability. In this regard, the stability of MNB-BECs at various temperatures and relative humidity levels needs to be studied along with their interrelationships with preservation performance. In general, the MNB-BEC approach appears to be an interesting multidisciplinary concept that combines colloidal science, gas–liquid interface engineering, and packaging for foods. Indeed, the possibility of replacing conventional packaging with an edible coating—from a passive barrier to an active one—might offer an avenue for the development of biodegradable and multifunctional packaging technologies, which will help reduce the problem of postharvest losses associated with conventional plastic packages.

## 9. Conclusions

The use of micro and nanobubbles in the development of biopolymer-based edible films is a potential approach for creating novel and sustainable postharvest preservative systems. Micro/nanobubbles can alter structural, interfacial, mechanical, gas transfer and moisture barrier properties of biopolymer films and offer prospects for encapsulation of bioactive compound and their sustainable release. Their large specific surface area, interfacial and gas transfer abilities of micro/nanobubbles may help in better management of oxygen, carbon dioxide and other gases surrounding the fresh produce due to lower respiration rates, delayed ethylene production and may cause prevention of microbial degradation.

The use of the MNB-BEC system will allow the possibility of creating an intermediate state between the traditional passive coatings and more advanced ones which are gas-sensitive and bioactive. It is possible that the synergetic effect of bubble surfaces and interaction of biopolymers will be able to solve the problems related to the poor mechanical barrier and uncontrolled release of active substance in conventional packaging. Nevertheless, it should be noted that the proposed technology is new concept and has not been developed yet with integration of biopolymers. However, the proposed concept of MNB-BEC may encounter several technical and scientific challenges. The primary issue can be related to the stability and retention of the nanobubbles during the mixture, deposition, application, drying and storage, is subjected to the changes or affected due to dissolution, movement and rupture of the micro/nano bubbles. Interdependence of the size, concentration, distribution of the micro/nano bubble resulted in the mechanical resistance, moisture and gas are difficult to predict at present. The controlled release of active compounds and gases demands controlled kinetic and diffusion studies. In addition, antimicrobial activity depends on the type of gas used and the process and cannot be extrapolated to other MNB compositions. Stability over time depending on the temperature and relative humidity conditions, as well as migration and safety of active ingredients, need to be systematically evaluated. Large-scale production and homogeneous inclusion of MNBs into the coating matrix may also raise issues. Therefore, research & experiment investigation, regulations and economic evaluation need to be performed in future for the validation of this proposed technology.

## Figures and Tables

**Table 1:** Evidence status of proposed functions and properties of MNB-BEC systems.

Proposed Function/Property	Existing Evidence	Status in MNB-BEC	Required Validation
Nanobubble stability in aqueous media	Established for MNB dispersions; stability depends on bubble size, gas type, surface charge, and liquid-matrix conditions [12,28].	Established principle; matrix-dependent	Measure bubble size distribution, number concentration, and stability in the coating formulation
High gas–liquid interfacial area	Established physical characteristic of micro/nanobubbles and related to their mass-transfer behavior [12].	Established principle	Quantify bubble size distribution and interfacial characteristics in the coating formulation
Gas transfer/mass transfer	Enhanced gas dissolution and mass-transfer behavior have been reported in liquid MNB systems [12,28].	Potential function; not established for dried MNB-BEC	Gas-transfer/permeability measurements
Bubble retention during film drying	Direct evidence exists for entrapped hydrogen micro/nanobubbles in gelatin-based films; however, retention is expected to be system-dependent [29].	Research gap for MNB-BEC systems	Microscopy/particle characterization before and after drying
Mechanical reinforcement/modification	Nanobubble-assisted incorporation of nanofillers has modified mechanical properties of gelatin films [30].	Matrix- and formulation-dependent	Tensile strength, elongation, Young’s modulus and rheology
Moisture-barrier improvement	Nanobubble-assisted gelatin films have shown changes in water-vapor permeability, including an increase rather than a universal improvement [30].	Hypothesis: direction is system-dependent	WVTR/WVP measurements
Oxygen-barrier modification	Bubble incorporation can theoretically alter diffusion pathways, but direct evidence for oxygen-barrier performance of MNB-BEC is limited.	Hypothesis	OTR measurements and oxygen-retention studies
Controlled active release	Controlled release is established for active edible/biopolymer packaging systems, but a specific MNB-mediated release mechanism in MNB-BEC remains conceptual.	Hypothesis	Release kinetics, diffusion modeling and storage studies
ROS-mediated antimicrobial activity	Antimicrobial/ROS-related effects have been reported for certain gas/MNB systems and MNB-assisted food applications; effects depend strongly on gas type and treatment conditions [31,32].	Conditional; gas/process-dependent	ROS characterization plus microbial assays
Shelf-life extension	MNB treatments have demonstrated preservation effects in some fresh-produce applications, but this evidence does not establish shelf-life extension by MNB-integrated edible coatings [31,32].	Hypothesis for MNB-BEC	Real-produce coating trials under controlled storage conditions

**Table 2:** Functional distinction among candidate gases.

Gas	Primary Role	Potential MNB-BEC Function	Major Concern
O_2_	Respiratory gas/oxidation	Modify local O_2_ environment	Excess O_2_ may accelerate oxidation
CO_2_	Respiration/metabolic atmosphere	Alter produce atmosphere; antimicrobial at sufficient concentrations	Concentration-dependent physiological effects
O_3_	Strong oxidant	Antimicrobial/decontamination	High reactivity, safety, sensory and residue concerns
H_2_	Redox-active gas	Potential antioxidant/redox modulation	Evidence for edible coating applications is limited

**Table 3:** Structure–Property Relationships in MNB-BEC Systems.

MNB Parameter	Expected Effect	Relevant Coating Property	Important Caveat
Bubble diameter	Changes surface-area/volume ratio and gas dissolution	Gas transfer, interfacial interactions	Effect depends on matrix
Bubble concentration/volume fraction	Changes dispersed-phase fraction and tortuosity	WVTR, OTR, tensile properties	Excess bubbles may create defects
Bubble size distribution	Determines uniformity of microstructure	Mechanical/barrier performance	Aggregation/coalescence can produce weak regions
Zeta potential	Indicates electrostatic stability in dispersion	Dispersion before film formation	Does not directly prove final-film adhesion
Polymer molecular weight	Affects chain entanglement	Strength, flexibility, diffusion	Matrix-specific
Polymer polarity/hydrophilicity	Affects gas/water interactions	WVTR and OTR	Hydrophilic polymers generally remain moisture-sensitive
Interfacial adhesion	Determines stress transfer	Tensile strength, elongation	Must be measured in final film
Bubble retention after drying	Determines whether bubbles remain	Porosity, barrier, gas storage	Requires experimental validation

**Table 4:** MNB-BEC systems and their potential characterization.

Indicator	Method	Standard	Expected Scope	Challenges
Bubble size/distribution	NTA/DLS/optical microscopy	ISO/validated method	Determine size distribution and population	Matrix interference
Bubble retention	NTA/optical imaging before–after coating	Proposed protocol	Quantify retention after drying	Bubble loss during sampling
Film morphology	SEM/cryo-SEM/confocal microscopy	ASTM/ISO where applicable	Determine pores, defects, and distribution	Sample preparation
Film thickness	Micrometer/profilometry	ASTM D6988 [82]	Uniformity and thickness	Surface irregularity
WVTR	Gravimetric method	ASTM E96 [83]	Moisture-barrier performance	RH dependence
OTR/CO_2_ permeability	Gas permeability analyzer	ASTM/ISO	Gas-transfer characteristics	Produce-specific requirements
Mechanical properties	Tensile testing	ASTM D882 [84]	Tensile strength and elongation	Humidity sensitivity
Active release	Diffusion/release kinetics	Validated protocol	Release profile and mechanism	Matrix complexity
Microbial activity	Plate count/validated assay	ISO methods	Antimicrobial potential	Gas-specific effects
Produce quality	Weight loss, firmness, color, TSS, respiration	Standard postharvest methods	Real-produce performance	Commodity variability

## Data Availability

No new data were created or analyzed in this study. Data sharing is not applicable to this article.
